# Case of Chronic Dysentery

**Published:** 1876-03

**Authors:** 


### CASE OF CHRONIC DYSENTERRY.

Dr. T. G. Thomas (*New York Medical Journal*) gives the following treatment:

On the 19th of September Dr. H. F. Walker anæsthetized the patient, and I proceeded to make a thorough examination of the rectum. After etherization, she was placed in the left lateral position, and, after stretching of the sphincter ani by the fingers, a long duck-bill speculum was introduced. This was held by my nurse exactly as in vaginal examinations, while by a depressor I pressed downward the anterior rectal wall. No one who has not examined the rectum in this way can imagine the facility with which the whole canal can be seen. In this instance it was perfectly exposed up the sigmoid flexure. I now cleansed it of all fecal matters by a long glass tube, so bent upon itself at its upper extremity as to throw a stream of water from a Davidson's syringe back toward the anus.

Throughout the whole extent of the intestine exposed to view the mucous membrane was seen swollen, œdematous, hanging in hæmorrhoidal masses, and studded with deep ulcers with grayish bottoms. It was greatly engorged, and presented that deep-red, almost violet, hue which is seen in the throat in cases of diphtheria.

On this occasion no application was made, and, as the anæsthetic had disturbed the patient's stomach and rendered her nervous, nothing more was done until the 30th of September. Then, ether being again administered by Dr. Walker and the bowel thoroughly cleansed, I wrapped a small piece of wet cotton around the end of a whalebone rod, and, dipping it in pure commercial nitric acid, lightly touched the swollen mucous membrane and all the ulcers intervening between the sigmoid flexure and the anus. No superfluous fluid was allowed to attach itself to the cotton, and the cauterization was nowhere so decidedly practiced as to render the occurrence of sloughing possible.

Upon recovery from the anæsthetic, a slight amount of pain only was complained of, and, writing of the subsequent effect, the patient says: "It soothed me, and I slept well. This was the first real respite which I had experienced in five years."

At this time the patient was confined to the milk-diet as much as possible, and limited as to exercise; but, as both these plans of treatment had been adopted, and had failed, before she came under my care, I did not deem it wise to press them too much upon her, for fear of disheartening her. This application proved of decided benefit, in diminishing the number of evacuations, the amount of blood passed, and the degree of pain experienced.

On the 6th of October, another application of nitric acid was made. This proved still more beneficial. The patient, in her written history, declares: "The second application improved me very decidedly." After it, the milk-diet was more strictly adhered to, and exercise was more restricted.

On the 11th of October, the third and last application was made. Dr. Walker and myself were then both struck by the great improvement in the appearance of the bowel. The ulcers had almost entirely disappeared; the mucous membrane was much less swollen, and the appearance of engorgement much modified. After this application, the milk-diet was strictly adhered to, and the patient for ten days confined to bed. The result of this application surprised me. Blood ceased to pass with the evacuations; these in three days became limited to one in twenty-four hours; all pain ceased; and the patient rapidly improved in general appearai ce, in flesh, and in spirits. "To-day," she writes, "October 26th, I feel that I am entirely relieved, having now for eight days had only one action in every twenty-four hours. All pain has left me. I am gaining flesh, color, appetite and spirits, and there is not even a trace of dysentery left."

